# Physical Training Reduces Chronic Airway Inflammation and Mediators of Remodeling in Asthma

**DOI:** 10.1155/2022/5037553

**Published:** 2022-10-20

**Authors:** Renilson Moraes-Ferreira, Maysa Alves Rodrigues Brandao-Rangel, Thiago Gonçalves Gibson-Alves, Anamei Silva-Reis, Victor Hugo Souza-Palmeira, Helida Cristina Aquino-Santos, Claudio Ricardo Frison, Luis Vicente Franco Oliveira, Regiane Albertini, Rodolfo P. Vieira

**Affiliations:** ^1^Federal University of São Paulo (UNIFESP), Post-graduation Program in Sciences of Human Movement and Rehabilitation, Santos, SP, Brazil; ^2^Unievangelica, Post-graduate Program in Human Movement and Rehabilitation and in Pharmaceutical Sciences, Anápolis, GO, Brazil; ^3^Brazilian Institute of Teaching and Research in Pulmonary and Exercise Immunology (IBEPIPE), São José dos Campos, SP, Brazil; ^4^Universidade Brasil, Post-graduate Program in Bioengineering, São Paulo, SP, Brazil

## Abstract

Several benefits of aerobic training for asthmatic patients have been demonstrated. However, its effects on systemic inflammation and on airway remodeling mediators and lung mechanics are unknown. This prospective study included 21 intermittent and mild asthma patients, and as primary outcomes, the evaluation of pro- and anti-inflammatory and pro- and antifibrotic mediators in exhaled breath condensate (EBC) and blood were performed, beyond the cell counting in blood and in induced sputum. Aerobic training was performed for 3 months, 3 times per week. Aerobic training increased the levels of anti-inflammatory cytokines and of antifibrotic mediators in the breath condensate: IL-1ra (*p* = 0.0488), IL-10 (*p* = 0.0048), relaxin-3 (*p* = 0.0019), and klotho (*p* < 0.0043), respectively. Similarly, in plasma, increased levels of IL-1ra (*p* = 0.0147), IL-10 (*p* < 0.0001), relaxin-3 (*p* = 0.004), and klotho (*p* = 0.0023) were found. On contrary, reduced levels of proinflammatory cytokines in the breath condensate, IL-1*β* (*p* = 0.0008), IL-4 (*p* = 0.0481), IL-5 (*p* < 0.0001), IL-6 (*p* = 0.0032), IL-13 (*p* = 0.0013), and TNF-*α* (*p* = 0.0001) and profibrotic markers VEGF (*p* = 0.0017) and TSLP (*p* = 0.0056) were found. Similarly, in plasma, aerobic training significantly reduced the levels of proinflammatory cytokines IL-1*β* (*p* = 0.0008), IL-4 (*p* = 0.0104), IL-5 (*p* = 0.0001), IL-6 (*p* = 0.006), IL-13 (*p* = 0.0341), and TNF-*α* (*p* = 0.0003) and of profibrotic markers VEGF (*p* = 0.0009) and TSLP (*p* < 0.0076). Fractional exhaled nitric oxide (FeNO) was reduced after the intervention (*p* = 0.0313). Regarding inflammatory cells in sputum, there was a reduction in total cells (*p* = 0.008), eosinophils (*p* = 0.009), and macrophages (*p* = 0.020), as well as of blood eosinophils (*p* = 0.0203) and lymphocytes (*p* = 0.0198). Aerobic training positively modulates chronic airway inflammation and remodeling mediators, beyond to improve systemic inflammation in intermittent and mild asthmatic patients.

## 1. Introduction

Asthma is a chronic inflammatory disease of the airways that affects approximately 272 million people worldwide [1]. Among several consequences of asthma, airway remodeling occurs, characterized by changes in the airway structures, including epithelial metaplasia, subepithelial fibrosis, angiogenesis, and thickening of smooth muscle [2]. This remodeling promotes irreversible loss of lung function beyond airway hyperresponsiveness and is associated with the severity of the disease [3].

The chronic inflammation of the airways is sustained and characterized by high levels of proinflammatory cytokines (IL-1*β*, IL-4, IL-5, IL-6, IL-13, and TNF-*α*), reduced levels of anti-inflammatory cytokines (IL-1ra and IL-10), and intense infiltration of inflammatory cells in the airways [4, 5]. This is accompanied by deregulation of the expression of fibrotic mediators, displaying high levels of profibrotic mediators, such as VEGF and TSLP and reduction of antifibrotic mediators (relaxin and klotho), generating a cycle of damage-repair responsible for the airway remodeling [4, 6, 7]. In addition, it was evidenced that asthmatics, particularly moderate, severe, and difficult to treat, have low-grade systemic inflammation and high levels of blood and lung eosinophils [8, 9]. Thus, it is essential to validate strategies that modulate these complex processes, once the available drugs reduce chronic inflammation, but not airway remodeling [3]. Therefore, as a nonpharmacological strategy, a growing number of evidence show that aerobic exercise presents great benefits by reducing airway inflammation and airway remodeling in a mice model of asthma [10–12]. However, no studies confirmed such findings in asthmatics, except to airway inflammation and airway hyperresponsiveness [13, 14]. Furthermore, previous studies have shown that physical exercise is potentially beneficial for asthmatic individuals, improving quality of life and tolerance to exertion and reducing asthma symptoms [10]. However, there is still divergence about the effectiveness of pulmonary rehabilitation for improvement of lung function of asthmatic individuals [15, 16].

Thus, we hypothesize that moderate intensity supervised aerobic training (SAT) performed 3 times per week for 3 months could reduce the levels of proinflammatory and profibrotic mediators while increase the levels of anti-inflammatory and antifibrotic mediators, reducing pulmonary and systemic inflammation in intermittent and mild asthmatic patients. Also, we hypothesize that the SAT can improve the lung mechanical function, quality of life, and physical capacity of asthmatic patients.

## 2. Material and Methods

### 2.1. Study Design and Participants

The study is a prospective clinical study. A control group was not included due to limitations found during the pandemic period for COVID-19, although it is proved that regular physical exercise provides numerous health benefits for nonasthmatics as well [17]. This research was carried out in the periods of June to December 2020 (24 patients, 11 completed) and February to July 2021 (22 patients, 9 completed), in this period occurred the pandemic of COVID-19, which imposed countless difficulties for the development of this study. Initially, 46 asthma patients were included in the study, but the ones who fulfilled the inclusion and exclusion criteria were only 21 patients with intermitted and mild asthma. All patients were diagnosed with asthma according to the criteria proposed by the Global Initiative for Asthma (GINA) and were examined by a pneumologist. The study was developed in the Laboratory of Pulmonary Immunology and Exercise (LABPEI), at the Federal University of São Paulo (UNIFESP). Inclusion criteria are as follows: (i) without neurological, respiratory, cardiac, and/or skeletal muscle diseases; (ii) without muscle, tendon, or skeletal limitations; (iii) clinical-drug treatment ≥ 6 months; (iv) no asthma crisis ≥ 30 days; (v); sign the free informed consent form; (vi) age between 20 and 60 years; and (vii) without SARS-CoV-2 infection (COVID-19). Exclusion criteria are as follows: (i) perform less than 75% of SAT sessions; (ii) seizures or drug exchange during the study; (iii) not follow the supervisor's guidance in SAT sessions; and (iv) SARS-CoV-2 infection (COVID-19) during the intervention period. All asthma patients signed the free and informed consent form, and the present study was approved (3.409.803) in the ethics committee of Federal University of São Paulo (UNIFESP). All measurements, described below, were performed before and after the 3-month period of SAT.

### 2.2. Evaluation of Fractional Exhaled Nitric Oxide (FeNO)

The FeNO was measured using the monitor NoBreath (Bedfont Scientific, UK) as a sensible biomarker of lung inflammation, and the results were expressed in parts per billion (ppb) [18]. The assessment was performed at baseline and after the intervention with the SAT, as it does not require effort for its correct execution; it was performed before and on the same day as the air-condensate collection and assessment of lung function and mechanics.

### 2.3. Evaluation of Inflammatory and Fibrotic Mediators in EBC

Exhaled breath condensate collection was performed through the tube RTube (Respiratory Research, USA), in which the patient breathed at a tidal volume for a period of 10-15 minutes [18]. The sample collected was stored at -86°C for the analysis of inflammatory (IL-1beta, IL-1ra, IL-05, IL-6, IL-10, and IL-13) and fibrotics (relaxin-3, klotho, VEGF, and TSLP) mediators through the ELISA technique, using commercial kits from R&D Systems (USA).

### 2.4. Evaluation of Lung Inflammation through the Induced Sputum

The collection of induced sputum follows the method described by Pavord et al. [19], with processing in a maximum of 1 hour, in which 2 ml of sputum was dissolved and centrifuged at 1000G for 7 minutes, at 4°C to separate the supernatant of the button cells, which was resuspended in 1 ml of PBS; an aliquot was used to count the total number of cells in the Neubauer chamber (hematocytometer) and to make the cytospin slide and stained with Diff Quick (Sigma-Aldrich, São Paulo, Brazil) for the differential cell count.

### 2.5. Evaluation of Whole Blood

The venous blood was collected in a tube containing EDTA K3 anticoagulant. Twenty-five microliters of blood was used to perform the complete blood analysis using the Sysmex XS-800i equipment, and the remained volume was centrifuged at 1800G, 7 minutes at 4°C, and the plasma was stored at -86°C to measure the levels of proinflammatory (IL-1*β*, IL-4, IL-05, IL-6, IL-13, and TNF-*α*) and anti-inflammatory (IL-1ra and IL-10) cytokines and profibrotic (VEGF and TSLP) and antifibrotic (relaxin-3 and klotho) mediators by ELISA technique, using commercial kits from R&D Systems (USA).

### 2.6. Clinical Characterization

On the second day, anthropometric (weight and height) and body composition were evaluated by Bioimpedance (Maltron 920-II-S, Maltron Inc., England) and inspiratory (MIP) and expiratory (MEP) muscle strength by manuvacuometer (MVD-300 V.1.1 Microhard System, Globalmed, Porto Alegre, Brazil); the general muscle strength was evaluated by hand grip dynamometer (Jamar®, Sammons Preston Rolyan, Bolingbrook, IL, USA); asthma symptoms were evaluated by the Asthma Control Questionnaire (ACQ7); the impact on quality of life was evaluated by the Asthma Quality of Life Questionnaire (AQLQ).

### 2.7. Evaluation of Lung Function and Mechanics

The lung function (spirometry method) and pulmonary mechanics (impulse oscillometry method; IOS Masterscreen, Jaeger, Germany) pre- and post-bronchodilator (Salbutamol 400mcg), following the recommendations [20]. Were the measurement of clinical parameters such as lung function forced vital capacity – predicted (FVC%), forced expiratory volume in 1° sec - predicted (FEV1%), FEV1/FVC%, forced expiratory volume in 3° sec (FEV3), forced expiratory volume in 6° sec (FEV6), peak expiratory flow - predicted (PEF%), maximal expiratory flow 25% - predicted (MEF25%), MEF50%, MEF75%, MEF25/75%, and pulmonary mechanics, respiratory system impedance – predicted (Z5%), total respiratory system resistance – predicted (R5Hz%), proximal airway resistance – predicted (R20Hz%), distal airway resistance (R5Hz-R20Hz), lung reactance (X5%), central resistance (RCentral), peripheral resistance (RPeripheral) and resonance frequency (RFres).

### 2.8. Evaluation of Functional Capacity

The functional capacity was assessed by the 6-minute walk test according to the recommendations of the American Thoracic Society (ATS) [20].

### 2.9. Intervention by Supervised Aerobic Training

Before starting each SAT session, we performed anamnesis on asthma symptoms (wheezing in the chest, cough, and shortness of breath), beyond the evaluation of heart rate, blood pressure, body temperature and oxygen saturation (SpO2%), and expiratory peak flow to assure patient safety. The first week of SAT was familiarization with 40-minute sessions, 5 initial minutes of warm-up, 30 minutes at intensity of 50% to 60% of the reserve heart rate (RHR), and the remaining 5 minutes for cooling.

The intervention was SAT for 12 weeks, performed on a treadmill, 3 times a week on a nonconsecutive day, each session consisted of 10 minutes of low-intensity warm-up, followed by 35 minutes at moderate intensity (70% to 80% of RHR), and finally 5 minutes of cooling [14, 21]. The training zone was based on RHR and the maximum heart rate was determined by the formula of Tanaka et al. [22]. In addition, the Borg effort perception scale in all aerobic training sessions was used, with optimal effort from 6 to 7 [13, 15]. All SAT sessions were accompanied by qualified physical education professionals.

### 2.10. Statistical Analysis

The data were analyzed using GraphPad Prism 8.0.1 software (California, USA). Data descriptions were made as mean ± standard deviation (M ± SD), median (interquartile 1st and 3rd), and difference of means (DM) and confidence interval at the 95% level (95% CI). The distribution of data normality was assessed using the Shapiro-Wilk test. Student's *t* test was used to compare data with normal distribution. One-way analysis of variance (ANOVA) (Bonferroni's) was used for parameters of lung function and mechanics (bronchodilator (BD) sensitivity before and after 12 weeks of aerobic training (pre-BD before vs. post-BD before and pre-BD after vs. post-BD after) and effect of 12 weeks of aerobic training (pre-BD before vs. pre-BD after and post-BD before vs. post-BD after)). Significance levels were adjusted to 5% (*p* < 0.05).

## 3. Results

Initially, 46 asthmatic patients were included, but only 21 (57.14%) met the study's criteria. From these 21 patients enrolled initially in the study, all have completed the study's protocol.

### 3.1. Effects of SAT on Clinical Characteristics of Asthma Patients

Table 1 describes and compares the clinical characteristics of asthmatic patients before and after the aerobic training program. A significant difference was found in the reduction of the FeNO (before = 41.41 ppb ± 39.16; after = 25.82 ppb ± 18.33; *p* < 0.05; DM: -15.59; 95% CI 1.58 to 29.59) and in the improvement in the hand grip strength right (DM: 2.94; 95% CI -5.58 to -0.30) and left (DM: 4.79; 95% CI -7.23 to -2.35).

### 3.2. Effects of SAT on Anti-Inflammatory and Antifibrotic Mediators in Asthma Patients

**Figure 1:**
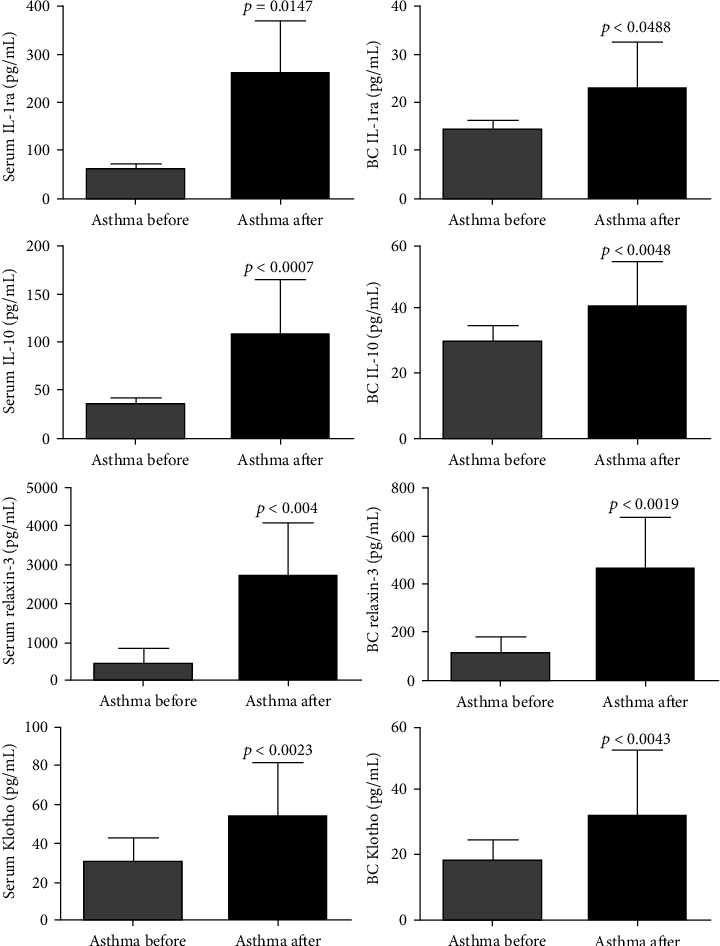
SAT increases anti-inflammatory and antifibrotic markers in the pulmonary airways and plasma of asthmatics. All 21 volunteers were evaluated for the plasma levels of anti-inflammatory and antifibrotic markers in plasma and in the lungs (breath condensate). Interleukin 1ra (IL1ra) and interleukin 10 (IL10). The figures lacking a symbol indicating statistical significance mean that no significance was found. BC = breath condensate.

Concerning the effects of SAT on airway inflammation and on mediators of lung fibrosis in patients with intermittent and mild asthma, it was found that SAT positively modulated airway inflammation and the remodeling biomarkers. After 12 weeks of SAT, there was an increase in the anti-inflammatory cytokines IL-1ra (DM: 8.44; 95% CI -16.82 to -0.06) and IL-10 (DM: 12.22; 95% CI -20.08 to -4.35) and an increase in the levels of antifibrotic biomarkers, relaxin-3 (DM: 335.5; 95% CI -500.4–-170.6) and klotho (DM: 13.24; 95% CI -21.68 to -4.79), which has been measured in the breath condensed. The same pattern of response was identified in plasma, higher levels of anti-inflammatory cytokines IL-1ra (DM: 200.7; 95% CI -363.4 to -37.96), IL-10 (DM: 46.52; 95% CI 59.67 to -33.37), and increased levels of antifibrotic biomarkers relaxin-3 (DM: 2235; 95% CI -3441 to -1029) and klotho (DM: 23.36; 95% CI -36.86 to -9.86) (Figure 1).

### 3.3. Effects of SAT on Proinflammatory and Profibrotic Mediators in Asthma Patients

**Figure 2:**
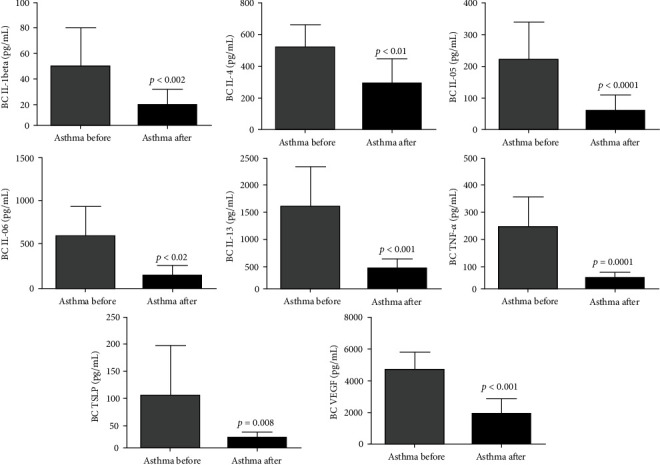
SAT reduces proinflammatory and profibrotic markers in the pulmonary airways of asthmatics. All 21 volunteers were evaluated for the pulmonary (breath condensate) levels of proinflammatory and profibrotic markers. Interleukin 1 beta (IL-1beta), interleukin 4 (IL-4), interleukin (IL-5), interleukin 6 (IL-6), interleukin 13 (IL-13), vascular endothelial growth factor (VEGF), tumor necrosis factor-alpha (TNF-*α*), and thymic stromal lymphopoietin (TSLP). The figures lacking a symbol indicating statistical significance mean that no significance was found. BC = breath condensate.

SAT reduced the proinflammatory cytokines responsible for the recruitment and maintenance of the chronic inflammatory process in the airways, IL-1*β* (DM: -29.91; 95% CI 14.33 to 45.48), IL-4 (DM: -151.7; 95% CI 1.569 to 301.8), IL-5 (DM: -162.3, 95% CI 105.7 to 219.0), IL-6 (DM: -169.9; 95% CI 66.44 to 273.4), IL-13 (DM: -1191; 95% CI 643.8 to 1739), and TNF-*α* (DM: -213.8; 95% CI 134.3 to 293.2) and the profibrotic biomarkers responsible for signaling in the airway remodeling process, VEGF (DM: -1406; 95% CI 604.3 to 2208) and TSLP (DM: -83.47; 95% CI 25.89 to 141.1) (Figure 2).

**Figure 3:**
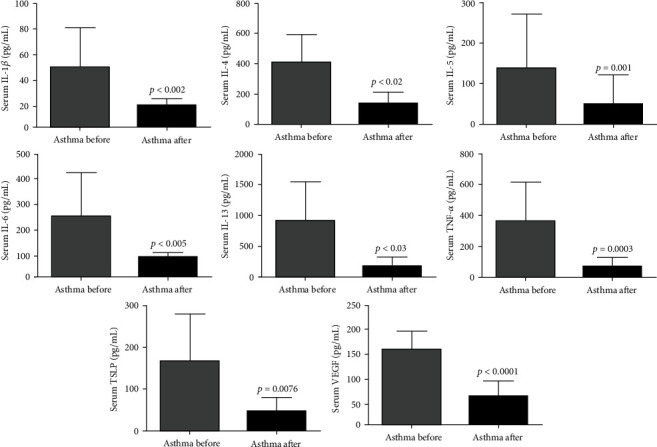
SAT reduces proinflammatory and profibrotic markers in the plasma of asthmatics. All 21 volunteers were evaluated for the plasma levels of proinflammatory and profibrotic markers. Interleukin 1 beta (IL-1beta), interleukin 4 (IL-4), interleukin (IL-5), interleukin 6 (IL-6), interleukin 13 (IL-13), vascular endothelial growth factor (VEGF), tumor necrosis factor-alpha (TNF-*α*), and thymic stromal lymphopoietin (TSLP). The figures lacking a symbol indicating statistical significance mean that no significance was found.

Similarly in plasma, after intervention with SAT, there was a significant reduction in proinflammatory cytokines IL-1*β* (DM: -51.99; 95% CI 23.46 to 80.51), IL- 4 (DM: -372.0; 95% CI 118.5 to 625.5), IL-5 (DM: -90.64; 95% CI 52.29 to 129.0), IL-6 (DM: -144.8; 95% CI 36.67 to 252.8), IL-13 (DM: -744.5; 95% CI 82.63 to 1406), and TNF-*α* (DM: -271.4; 95% CI 156.2 to 386.5) and of the VEGF (DM: -193.2; 95% CI 92.18 to 294.2) and TSLP (DM: -121.3; 95% CI 41.05 to 201.6) profibrotic biomarkers (Figure 3).

### 3.4. Effects of SAT on Pulmonary and Systemic Inflammatory Response in Asthma Patients

**Figure 4:**
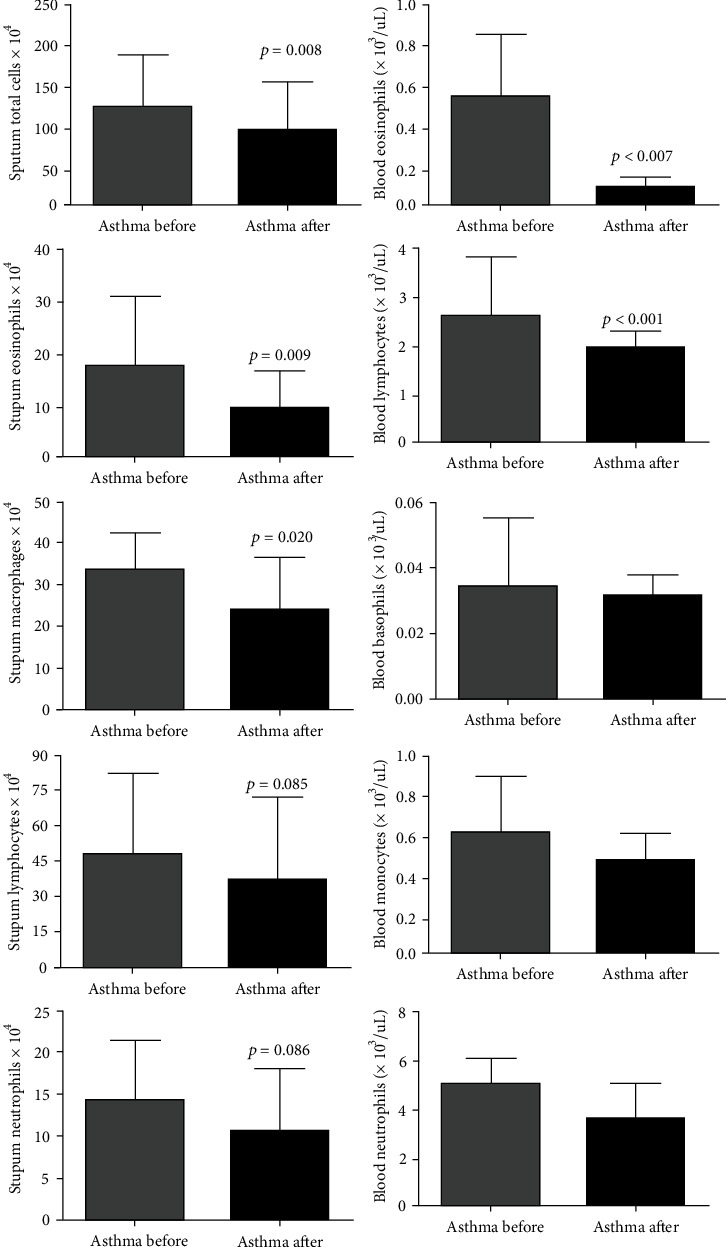
SAT reduces the number of inflammatory cells in the sputum and blood of asthmatics. The figures lacking a symbol indicating statistical significance mean that no significance was found.

The positive modulation of mediators of inflammation and pulmonary fibrosis biomarkers by SAT was accompanied by a reduction in the infiltration of inflammatory cells in the airways of asthmatic patients. The results demonstrated a reduction in the number of total cells (DM: -31.50; 95% CI 7.13 to 55.86), eosinophils (DM: -7.90; 95% CI 1.69 to 14.12), and macrophages (DM: -9.48; 95% CI 0.55 to 18.40) in induced sputum. No differences in the number of neutrophils (DM: -3.77; 95% CI -1.98 to 9.52) and lymphocytes (DM: -11.32; 95% CI -5.90 to 28.55) were observed. The analysis of inflammatory cells in the blood showed a reduction in the number of lymphocytes (DM: -0.36; 95% CI 0.02 to 0.71) and eosinophils (DM: -0.08; 95% CI 0.004 to 0.174), with no difference in neutrophils (DM: -0.15; 95% CI -0.56 to 0.87), monocytes (DM: -0.07; 95% CI -0.02 to 0.17), and basophils (DM: -0.002; 95% CI -0.006 to 0.012) (Figure 4).

### 3.5. Effects of SAT on Quality of Life, Aerobic Capacity, and Respiratory Muscle Strength in Asthma Patients

**Figure 5:**
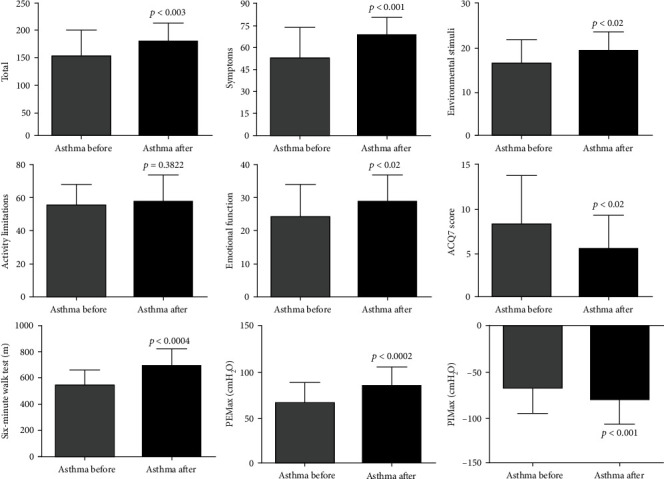
SAT improves quality of life, asthma symptoms, and aerobic capacity and peripheral and respiratory muscle strength in asthmatics. All 21 volunteers were evaluated for quality of life, asthma symptoms, aerobic capacity, and peripheral and respiratory muscle strength. Asthma Quality of Life Questionnaire (AQLQ), subdivision of AQLQ (symptoms, activity limitations, emotional function, and environmental stimulus), Asthma Control Questionnaire 7 (ACQ7), maximal expiratory pressure (MEP), and maximal inspiratory pressure (MIP). The figures lacking a symbol indicating statistical significance mean that no significance was found.

Regarding the secondary outcomes, 12 weeks of SAT resulted in improvement of total quality of life (DM: 25.06; 95% CI -42.02 to -8.09), symptoms (DM: 15.72; 95% CI -25.11 to -6.33), emotional function (DM: 4.31; 95% CI -8.0 to -0.61), and environmental stimulation (DM: 2.76; 95% CI -5.45 to -0.07), but not in physical limitations (DM: -1.35; 95% CI -7.94 to 10.64). Twelve weeks of SAT also improved asthma control (DM: -3.42; 95% CI 0.46 to 6.38), aerobic capacity (DM: 142.7; 95% CI -209.5 to -75.94), maximum inspiratory (MIP (DM: -12.40; 95% CI 4.71 to 20.09)), and expiratory (MEP (DM: 18.71; 95% CI -27.54 to -9.870)) pressure (Figure 5).

### 3.6. Effects of SAT on Lung Function and Lung Mechanics in Asthma Patients

**Figure 6:**
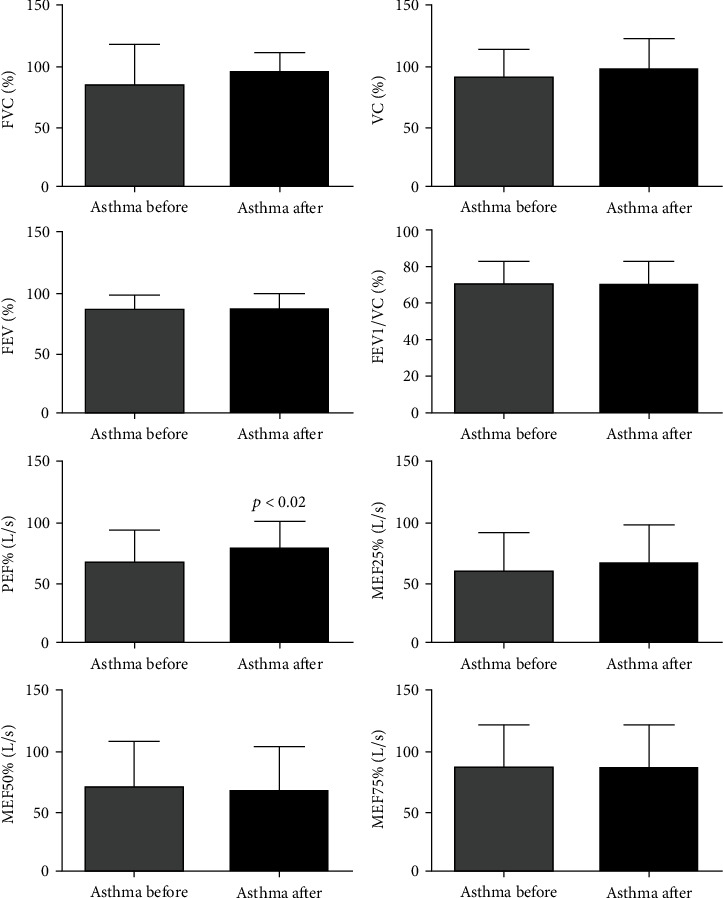
Effects of SAT on lung function of asthmatics. All 21 volunteers were evaluated for lung function. Forced vital capacity (FVC), vital capacity (VC), forced expiratory volume in the first second (FEV1), FEV1/FVC ratio, peak expiratory flow (PEF), maximum expiratory flow 25% (MEF25%), maximum expiratory flow 50% (MEF50%), and maximum expiratory flow 75% (MEF75%). Graphs lacking a symbol indicating statistical significance mean that no significance was found on lung function parameters after 12 weeks of aerobic training (pre-BD before vs. post-BD before).

**Figure 7:**
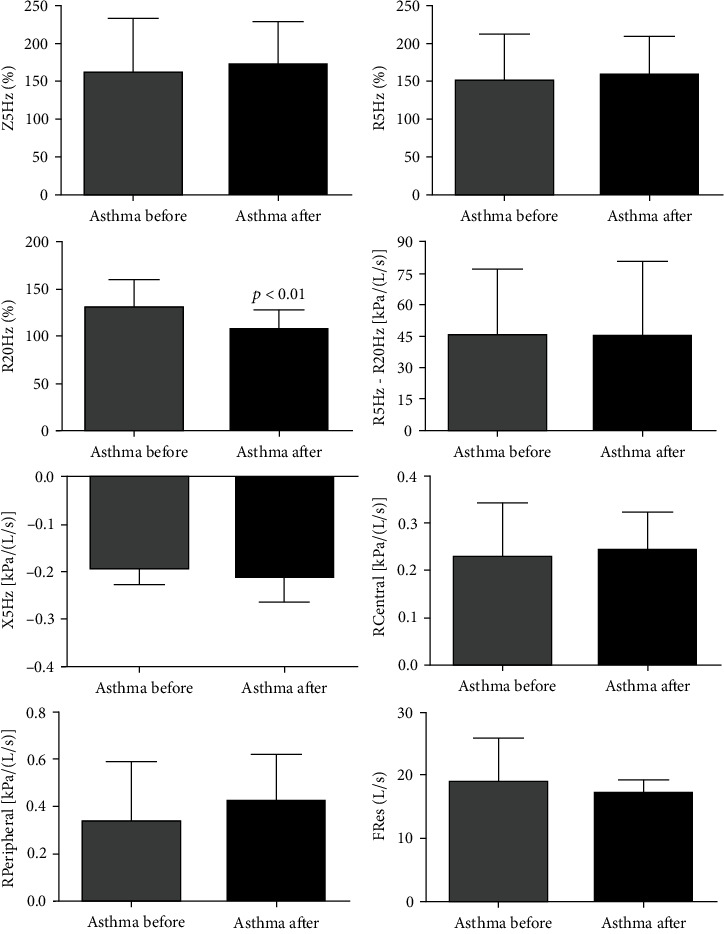
Effects of SAT on lung mechanics of asthmatics. All 21 volunteers were evaluated for lung mechanics. Respiratory impedance at 5 Hz (Z5Hz%), resistance of whole respiratory system (R5Hz%), resistance of proximal airways (R20Hz%), resistance of distal airways (R5Hz-R20Hz%), reactance of respiratory systems (X5Hz), central resistance (RCentral), peripheral resistance (RPeripheral), and resonance frequency (RFres). The figures lacking a symbol indicating statistical significance mean that no significance was found. Graphs lacking a symbol indicating statistical significance mean that no significance was found on lung mechanics parameters after 12 weeks of aerobic training (pre-BD before vs. post-BD before).

The data on pulmonary function parameters (FVC%, FEV1%, FEV1/FVC%, FEV3, FEV6, MEF25%-75%, and MEF25/75%) revealed no changes, except for PEF% (*p* < 0.013) in patients with intermittent and mild asthma after SAT (Figure 6). Concerning the pulmonary mechanics (Z5Hz%, R5Hz%, R5Hz-R20Hz, RCentral, RPeripheral, X5Hz, and RFres), no effects were observed, except for R20Hz% (*p* < 0.016) in patients with intermittent and mild asthma after SAT. Furthermore, it was not observed any difference in response to bronchodilators in both periods, demonstrating that the patients were well controlled (Figure 7).

## 4. Discussion

The present study confirmed the hypothesis that moderate-intensity SAT performed 3 times per week reduces chronic airway inflammation beyond to reduce the levels of pulmonary and systemic profibrotic biomarkers, increasing the levels of antifibrotic biomarkers in asthmatic patients in early stages of disease, notably intermittent and mild asthmatics. In addition, the study also confirmed the sensitivity of FeNO in detecting the effectiveness of SAT in decreased pulmonary inflammation. Likewise, SAT promoted a reduction in eosinophil and macrophage accumulation in the airways and a reduction in the blood eosinophils and lymphocytes of asthmatic patients. As secondary findings, the study demonstrated that SAT improved quality of life, asthma symptom control, exercise tolerance with increased aerobic capacity, and maximum respiratory muscle strength. On the other hand, 12 sessions of SAT performed 3 times per week improved PEF% and R20Hz%, while the other parameters of lung function and mechanics remained unchanged in patients with intermittent and mild asthma. Of note, this was the first study investigating the effects of SAT on lung mechanics.

As a gold target, it is essential to inhibit the pathophysiological mechanisms that leads to airway remodeling in asthmatic patients, once it means prevention of several structural injury, such as epithelial damage and ciliary dysfunction, hypertrophy and hyperplasia of the goblet cells and bronchial smooth muscle, increased thickness of the reticular lamina and reticular basement membrane, increased mucus synthesis and increased deposition of extracellular matrix proteins (collagen fibers, elastic fibers, proteoglycans, and laminins), increased vascularization, and increased number and activation of subepithelial myofibroblasts and airway fibroblasts [3, 23]. This whole set of alterations leads to tissue dysfunction, exacerbations, hyperresponsiveness, and physical limitations for the asthmatic patient [3, 23] and constitutes the biggest challenge in the treatment for asthma. Herein, although this study has not evaluated the thickness of airway wall by computerized tomography (as a proof of airway remodeling), the improvement induced by SAT on R20Hz% may represent a functional improvement in airway remodeling, which can be reinforced by the reduction in the profibrotic biomarkers and increases in antifibrotic biomarkers in the lungs as well as systemically [2–8]. In addition, preclinical studies using asthma model have pointed out that aerobic exercise was capable to inhibit [16] and reverse [10] airway remodeling. However, we assume the limitation of the present study in affirm that SAT may improve airway remodeling in intermittent and mild asthmatic patients.

Previous studies have shown that aerobic exercise inhibits [10, 16] and reverses the process of inflammation and airway remodeling in mouse models of asthma, being mediated by increased expression of the anti-inflammatory cytokines IL-10 and IL-1ra, with a reduction in proinflammatory Th2 cytokines, such as IL-4, IL-5, and IL-13, which plays a key role in the maintenance of chronic inflammation and airway remodeling [2, 3, 5]. In this way, the present study was the first to investigate the effects of SAT on these classical Th2 cytokines, demonstrating a significant reduction on the pulmonary and systemic levels of IL-4, IL-5, and IL-13. Furthermore, the present study also showed that SAT induced a strong anti-inflammatory response, denoted by increased levels of IL-10 and IL-1ra in the lungs and systemically. In addition, another clinical study, which evaluated among other parameters in moderate and severe asthmatic patients, the effects of aerobic exercise on blood proinflammatory cytokines/chemokines (IL-5, IL-6, IL-8, and MCP-1/CCL2) and on the anti-inflammatory (IL-10), found that aerobic exercise only reduced the levels of IL-6 and MCP-1/CCL2 [13]. A possible explanation for this modest anti-inflammatory effect may be explained by the fact that such study submitted the asthmatic patients for aerobic training only twice a week, only 25 minutes of effective exercise, at high intensity, plus a yoga session as well [13]. In this context, we can hypothesize that the volume of exercise was not enough to induce the anti-inflammatory effects observed in the present study. However, we reinforce that this is the first study demonstrating in humans that SAT performed at a volume of 3 times per week and with 40 minutes per session reduces pulmonary and systemic Th2 response in intermittent and mild asthmatic patients. Beyond Th2 cytokines, SAT also reduced the levels of other potent proinflammatory cytokines (IL-1*β*, IL-6, and TNF-*α*) which also plays a key role in asthma pathophysiology and progression, accounting to disease severity [24]. Importantly, such positive immunomodulatory effects of SAT on humoral mediators were also followed by reduced accumulation of inflammatory cells in the airways, as denoted in the analysis of induced sputum, and by reduced systemic eosinophilic inflammation, which are highlights of progression and of the most severe forms of asthma [8, 9].

For the first time, the effects of SAT in the markers directly involved in structural changes of the airways in asthmatic patients were investigated, and our data showed that SAT reduced the levels of profibrotic mediators VEGF and TSLP while increased the levels of antifibrotic mediator's relaxin-3 and klotho, both in the lungs and in the plasma. So, such findings demonstrate that SAT inhibited the immune response underlying the airway remodeling. It is known that VEGF increases the differentiation and proliferation of fibroblasts and myofibroblasts, inducing the synthesis of exaggerated amounts of extracellular matrix proteins, such as collagen and elastic fibers, proteoglycans, and laminins [7]. In addition, TSLP maintains inflammation and can aggravate remodeling by activation of specific pathways that promote crosstalk between airway epithelial cells and fibroblasts [23]. On the other hand, relaxin-3 has antifibrotic effect by inhibiting growth factors and seems to reverse the fibrosis process in the airways of a mouse model of asthma [25]. In this way, another protein with antifibrotic properties, named klotho, which protects the lungs against oxidative damage and apoptosis, also inhibits the fibrotic response by suppressing the expression of VEGF and TGF-*β*1/Smad3 [4].

Another important aspect of chronic airway inflammation and remodeling in asthmatic patients is the excessive infiltration of inflammatory cells into the subepithelial tissue, causing local tissue/cell damage and the release of many proinflammatory and profibrotic mediators, maintaining the damage-repair cycle [26]. In a mouse model of asthma, it has been already shown that aerobic exercise reduces the infiltration of macrophages and eosinophils in the airways [13], findings that are confirmed by the data presented in this clinical study, in which SAT reduced eosinophil and macrophage infiltration into the airways. In addition, the present study also demonstrated that SAT reduced the number of eosinophils and lymphocytes in the blood, which point out the potential benefit of SAT in asthma scenario, in which increased systemic inflammation is associated with exacerbations and poor prognosis [8, 9].

Furthermore, all immunological effects observed in the present study may justify the improvement in asthma symptoms and quality of life observed as well. Of note, it has been described those structural changes, inflammation mediated by cytokines and inflammatory cells in the airways, are determinant aspects in the intensity of symptoms and exacerbations in asthmatic patients [27]. In this context, a very important biomarker of asthmatic inflammation and exacerbation is fractional FeNO, which was elevated in intermittent and mild asthmatic patients and reduced after the intervention with SAT. Previous studies have shown similar results after aerobic exercise with moderate and severe persistent asthma [14]. These results were accompanied by the improvement in exercise tolerance through the improvement in aerobic capacity, maximum respiratory, and peripheral muscle strength observed after 12 weeks of SAT, reinforcing the findings from previous studies that pointed out to beneficial effects of regular aerobic exercise to asthmatic patients [15].

Regarding lung function and mechanics, the systematic review by Ram et al. [15] showed improvement in cardiopulmonary fitness without altering pulmonary function, similar to the findings of the present study. Additionally, a meta-analysis [28] reported that exercises based on pulmonary rehabilitation program improved PEF, as observed in the present study. On the other hand, a study by Eichenberger et al. [29] observed an improvement in FEV1, but not on other parameters of lung function in asthmatic patients. In the present study, we observed only an improvement in the PEF, but not in other parameters of lung function. It is difficult to compare with previous studies due to the heterogeneity of interventions and sample phenotype, but the studies included by Ram et al. [15] were more likely to ours, in which aerobic exercise lasting 20 to 30 minutes was performed two to 3 times a week. Two factors that may be determinant for the findings of our study are that the patients included have controlled asthma for at least 30 days and are classified as having intermittent and mild persistent asthma.

## 5. Conclusion

Our findings show an unprecedented beneficial effects for intermittent and mild asthmatics, with the inhibition of the immunological mechanisms involved in the airway inflammation and remodeling in early stages of disease and intermittent and mild asthma. So, we conclude that SAT improves quality of life and respiratory muscle strength and reduces pulmonary and systemic inflammation, beyond to inhibit the profibrotic pathway, suggesting an action in decelerating the progression of airway remodeling, a feature to be investigated in further studies.

## Figures and Tables

**Table 1:** Clinical characteristics of asthmatic patients before and after the intervention.

Parameters	Before SAT	After SAT	*p* value
Age (years)	35.95 ± 15.59	36.19 ± 15.65	0.6339
Sex (M/F)^#^	9/12	—	—
Weight (kg)	84.47 ± 18.27	84.66 ± 18.39	0.9349
Height (m)	1.67 ± 0.08	1.67 ± 0.08	0.7766
BMI (kg/m^2^)	30.24 ± 6.43	30.23 ± 6.29	0.9740
Systolic blood pressure (mmHg)	120.62 ± 15.15	120.05 ± 14.51	0.5144
Diastolic blood pressure (mmHg)	80.70 ± 10.69	97.81 ± 10.97	0.8846
Heart rate (bpm)	81.2 ± 5.61	80.7 ± 4.57	0.4424
Fat mass (%)	38.57 ± 8.02	35.89 ± 10.40	0.3916
Fat free mass (%)	61.43 ± 8.02	64.11 ± 10.40	0.3916
SpO2%	97.81 ± 0.60	98.05 ± 0.39	0.0563
Hand grip strength right (kg)	34.69 ± 11.48	37.63 ± 10.50	0.0166
Hand grip strength left (kg)	32.08 ± 10.01	36.88 ± 10.02	0.0010
FeNO (ppb)	41.41 ± 39.16	25.82 ± 18.33	0.0313
GINA classification^∗^	1 (1–2)	—	—

BMI: body mass index; SpO2%: pulp oxygenation level; ACQ7: Asthma Control Questionnaire 7; GINA: Global Initiative for Asthma. Data are described in mean and standard deviation (mean ± SD). ^∗^Data in median, 1st, and 3rd interquartile. ^#^Data in absolute numbers (*n*).

## Data Availability

All raw data will be freely available upon request to corresponding author, under a reasonable justification.

## Additional Points

*Highlights*. (i) This study shows for the first time that aerobic exercise induces the release of anti-inflammatory cytokines, beyond to dampen the proinflammatory cytokines, systemically and also in the lungs. (ii) This study shows for the first time that aerobic exercise induces the release of antifibrotic proteins, beyond to dampen the profibrotic proteins, systemically and also in the lungs. (iii) This study shows for the first time that aerobic exercise reduces the resistance of the respiratory system, suggesting that aerobic exercise may reduce the airway remodeling.
